# Analysis of perioperative corticosteroid therapy in children undergoing cardiac surgery: A systematic review and meta‐analysis

**DOI:** 10.1002/clc.24018

**Published:** 2023-04-26

**Authors:** Daliu Chen, Yongchun Du

**Affiliations:** ^1^ Gastrointestinal Surgery Chunzhou County Hospital of Huai'an City Huai'an China; ^2^ Pediatric Surgery Huai'an Maternal and Child Health Care Hospital China

**Keywords:** cardiac surgery, cardiovascular events, corticosteroids, pediatric

## Abstract

The advantages and disadvantages of using corticosteroids in children undergoing cardiac surgery is still contentious. To examine how perioperative corticosteroids affect postoperative mortality and clinical outcomes in pediatric cardiac surgery with cardiopulmonary bypass (CPB). We used MEDLINE, EMBASE, and the Cochrane Database to conduct a comprehensive search up through January 2023. Children aged 0–18 undergoing cardiac surgery were included in the meta‐analysis of randomized controlled studies comparing perioperative corticosteroids with other therapeutic therapies, placebo, or no treatment. All‐cause hospital mortality was the primary endpoint of the study. Hospitalization duration was a secondary result. The Cochrane Risk of Bias Assessment Tool was used to evaluate the research quality. Ten trials and 7798 pediatric participants were included in our analysis. Children taking corticosteroids had no significant difference in all‐cause in‐hospital mortality using a random‐effect model with relative risk (RR) = 0.38, 95% confidence interval (CI) = 0.16–0.91, *I* ^2^ = 79%, *p* = .03 for methylprednisolone and RR = 0.29, 95% CI = 0.09–0.97, *I* ^2^ = 80%, *p* = .04. For the secondary outcome, there was a significant difference between the corticosteroid and placebo groups, with pooled standard mean difference (SMD) = −0.86, 95% CI = −1.57 to −0.15, *I* ^2^ = 85%, *p* = .02 for methylprednisolone and SMD = −0.97, 95% CI −1.90 to −0.04, *I* ^2^ = 83%, *p* = .04 for dexamethasone. Perioperative corticosteroids may not improve mortality, but they reduce hospital stay compared to placebo. Further evidence from randomized controlled studies with larger samples is required for approaching at a valid conclusion.

## Highlights

- The role of perioperative corticosteroids in preventing all‐cause mortality events in pediatric cardiac surgery patients is still doubtful.
- This systematic review and meta‐analysis sought to determine the effect of corticosteroids on mortality during cardiopulmonary bypass (CPB)‐assisted pediatric heart surgery.
- The administration of perioperative corticosteroids may not significantly improve the clinical outcomes of all‐cause mortality and length of hospital stay in children undergoing heart surgery.

## Graphical abstract

Perioperative corticosteroid therapy in children undergoing cardiac surgery. Perioperative corticosteroids may not improve morality, but they reduce hospital stay compared to placebo.

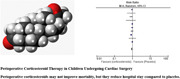

## 1 INTRODUCTION

Cardiac surgery in children, also known as pediatric cardiac procedures or pediatric cardiac surgery, is used to correct congenital heart problems like atrial septal defects, ventricular septal defects, atrioventricular canal canals, transposition of the great arteries, tetralogy of Fallot, and others. To accomplish this, the heart must be stopped and the chest cavity must be opened (a process known as cardiopulmonary bypass or CPB). It is a procedure required for the vast majority of pediatric patients undergoing corrective or palliative congenital heart surgery.

CPB may play a role in a complicated pathophysiological process due to exposure to the artificial interface of CPB circuits, hemodilution, hypothermia, ischemia/reperfusion of vital organs, and other considerations. In this process, the inflammatory response is overstimulated, leading to the production of inflammatory cytokines and the clustering of neutrophils.^1^, ^2^

In spite of advancements in perfusion techniques and the availability of alternative drugs to reduce the inflammatory response that occurs during CPB, corticosteroids have been the therapy of choice for nearly 50 years. Yet, there are currently ongoing controversies in this field.^3^, ^4^, ^5^ Anti‐inflammatory corticosteroids work by blocking the production of inflammatory molecules including endotoxin and cytokines.^6^, ^7^, ^8^ In addition, they may directly work as a supplementary therapy for adrenal insufficiency in neonates and infants brought on by CPB. This is because CPB causes the adrenal glands to become underdeveloped.^9^, ^10^, ^11^ Nevertheless, corticosteroids have been linked to a number of potential side effects, including hyperglycemia, poor wound healing, infections, and poor neurodevelopmental outcomes.^12^, ^13^, ^14^, ^15^ The effect of perioperative corticosteroids on inflammatory biomarkers, clinical outcomes, and adverse events following congenital heart operations have been the subject of a number of limited studies that have been randomized and controlled. These studies have been conducted on pediatric patients.^16^, ^17^, ^18^, ^19^, ^20^, ^21^, ^22^, ^23^, ^24^, ^25^ In spite of this, the outcomes were contradictory, which is what led to the debate on the use of perioperative corticosteroids. Therefore, the risk/benefit analysis of perioperative corticosteroids should be reevaluated in light of the findings of the recent studies, which showed an increase in comorbid conditions rather than benefits for clinical outcomes in lower‐risk patients. Therefore, it is important to undertake a thorough literature evaluation to weigh the benefits and drawbacks of using corticosteroids during surgery. In turn, this assessment can direct decisions on perioperative corticosteroids in pediatric heart surgery. Therefore, the main objective of this study is to evaluate whether perioperative steroids is beneficial for pediatric surgery or not. We conducted a comprehensive review of the literature and a meta‐analysis that was restricted to randomized controlled trials to examine the effects of perioperative corticosteroids: Dexamethasone and Methylprednisolone on the outcomes of patients undergoing pediatric cardiac surgery (Randomized Clinical Trials [RCTs]).

## 2 MATERIALS AND METHODS

### 2.1 Search strategy

Articles published in English exclusively between 2000 and January 2023 were retrieved from Medline, Embase, and the Cochrane Database. Independently, two researchers (D. C. and Y. D.) did the search and reviewed the bibliographies to find potentially relevant papers. The search terms used were: “cardiac surgery,” “coronary artery bypass,” “CPB,” “pediatric or children or neonatal cardiac surgery,” “corticosteroids,” “dexamethasone,” “methylprednisolone,” “all‐cause mortality,” “Length of hospital stay.”

### 2.2 Eligibility criteria

The two researchers referred to in the search procedure (D. C. and Y. D.) evaluated the titles and abstracts of the retrieved publications based on the following inclusion criteria. (i) published in English; (ii) conducted randomized clinical trials; (iii) included patients <18 years of age; (iv) included patients scheduled for congenital heart surgery; and (v) administered perioperative steroids: Dexamethasone and Methylprednisolone and included results for the primary outcome in‐hospital mortality and length of hospital stay. The research excluded papers that did not report either the primary or secondary outcomes, or that reported all outcomes as the median and interquartile range.

### 2.3 Data extraction and outcome measures

This review was conducted in accordance with the most recent edition of the Cochrane Handbook for Systematic Reviews of Intervention standards.^26^ A predesigned data collecting form was utilized to retrieve data from the primary research (Table 1). Two investigators (D. C. and Y. D.) extract the data independently. The major outcome measure was in‐hospital mortality resulting from any cause. The length of the patient's stay in the hospital was one of the secondary outcomes.

**Table 1:** Brief summary of included trials.

Study ID and year	Type of study	Journal of publication	Participants	Drug used	Control	Groups		Dosing regimen	Endpoints
						Intervention	Control		
Mott et al.^16^	Single‐center randomized double‐blind placebo‐controlled trial	*Journal of the American Collège of Cardiology*	Children undergoing cardiac surgery	Methylprednisolone	Placebo	126	120	Methylprednisolone (1 mg/kg)	Primary endpoint: all‐cause mortality Secondary endpoint: length of stay in hospital
Checchia et al.^17^	Prospective, randomized, double‐blind study	*Critical Care Medicine*	Children undergoing cardiac surgery	Dexamethasone	Placebo	15	13	Dexamethasone, 1 mg/kg	Primary endpoint: all‐cause mortality Secondary endpoint: length of stay in hospital
Heying et al.^18^	Prospective double‐blind randomized study	*The Annals of Thoracic Surgery*	Children undergoing cardiac Surgery	Dexamethasone	Placebo	11	9	Dexamethasone, 1 mg/kg	Primary endpoint: all‐cause mortality Secondary endpoint: length of stay in hospital
Pasquali et al.^19^	Multicenter observational analysis	*Pediatrics*	Neonates Undergoing cardiac surgery	Methylprednisolone	Placebo	1972	1700	Methylprednisolone (30 mg/kg)	Primary endpoint: all‐cause mortality Secondary endpoint: length of stay in hospital
Bunge et al.^20^	Multicenter, double‐blind, placebo‐controlled, randomized trial	*American Heart Journal*	Children undergoing cardiac surgery	Dexamethasone	Placebo	421	401	Dexamethasone, 1 mg/kg	Primary endpoint: all‐cause mortality Secondary endpoint: length of stay in hospital
Suominen et al.^21^	Single‐center randomized double‐blind placebo‐controlled trial	*The Annals of Thoracic Surgery*	Neonates undergoing cardiac surgery	Methylprednisolone	Placebo	20	20	Methylprednisolone 2 mg/kg	Primary endpoint: all‐cause mortality Secondary endpoint: length of stay in hospital
Graham et al.^22^	Two centers double‐blind randomized controlled trial	*Journal of the American College of Cardiology*	Neonates undergoing cardiac surgery	Methylprednisolone	Placebo	95	81	Methylprednisolone (30 mg/kg)	Primary endpoint: all‐cause mortality Secondary endpoint: length of stay in hospital
Lomivorotov et al.^23^	Multicenter randomized trial	*Journal of the American Medical Association*	Infants younger than 12 months undergoing cardiac surgery	Dexamethasone	Placebo	200	194	1 mg/kg of dexamethasone	Primary endpoint: all‐cause mortality Secondary endpoint: length of stay in hospital
Hill et al.^24^	Multicenter, prospective, randomized, placebo‐controlled, registry‐based trial	*The New England Journal of Medicine*	Infants <1 year of age undergoing heart surgery	Methylprednisolone	Placebo	601	599	Methylprednisolone (30 mg/kg of body weight)	Primary endpoint: all‐cause mortality Secondary endpoint: length of stay in hospital
Townsley et al.^25^	Multicenter (24 sites), prospective, randomized, placebo‐controlled trial	*Journal of Cardiothoracic and Vascular Anaesthesia*	Infants <1 year of age undergoing heart surgery	Methylprednisolone	Placebo	601	599	30 mg/kg of prophylactic methylprednisolone	Primary endpoint: all‐cause mortality Secondary endpoint: length of stay in hospital

### 2.4 Risk of bias assessment

The quality assessment of included studies was conducted using the “risk of bias” table in Review Manager (REVMAN) software (version 5.3; The Nordic Cochrane Center).^27^ Random sequence creation, allocation concealment, blinding of participants and personnel, blinding of outcome assessment, inadequate outcome data, selective reporting, and other sources of bias were catalogued in this table. Using this table, we were able to assign a score of “low,” “high,” or “uncertain” to each parameter in our quality assessment of the research. Two different investigators (D. C. and Y. D.) carried out the investigation separately.

### 2.5 Statistical analysis

REVMAN software (version 5.3; The Nordic Cochrane Center) was deployed for all analyses. For dichotomous and continuous outcome data, the relative risks (RRs) or weighted mean differences (WMDs) and their respective 95% confidence intervals (CIs) were calculated. Both *Χ* ^2^ and *I* ^2^ statistics were employed to evaluate the heterogeneity of the study. I‐squared values of 25%, 50%, and 75% were defined as low, middle, and high heterogeneity thresholds, respectively, and a *p*‐value <0.1 was considered indicative of heterogeneity.^28^ As there was high heterogeneity across the included papers, the random effects model was mostly utilized throughout all analyses. A funnel plot was used to determine publication bias. *p* < .05 was regarded as statistically significant.

## 3 RESULTS

### 3.1 Study characteristics and extraction

Supporting Information: Figure 1 displays the PRISMA chart for study selection. Through an exhaustive search of online resources, a total of 427 studies were identified. The abstracts and titles of 171 studies were reviewed after deleting duplicates. Only 43 studies were eligible for full‐text review. Based on the PICOS criteria,^29^ 10 publications were ultimately included in this meta‐analysis that reported the results of administration of perioperative steroids: Dexamethasone and Methylprednisolone in pediatric patients. Table 1 displays the major characteristics of all included trials involving children undergoing cardiac surgery. Six articles assessed the effects of corticosteroid: Methylprednisolone,^16^, ^19^, ^21^, ^22^, ^24^, ^25^ while four articles assessed the efficacy of corticosteroid: Dexamethasone.^17^, ^18^, ^20^, ^23^

### 3.2 Assessment of risk of bias and publication bias

**Figure 1:**
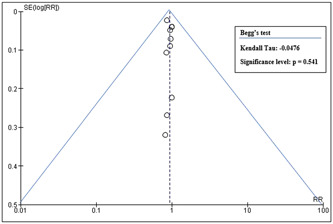
Funnel plot for publication bias.

Six of ten included studies showed a low risk of bias, while two had a substantial risk due to randomization process and bias in selection of the reported results. The other two studies exhibited high risk due to bias arising from the randomization process and bias in the selection of reported results as shown in risk of Bias summary (Supporting Information: Figure 2) and risk of Bias graph (Supporting Information: Figure 3). Figure 1 displays the funnel plot, which demonstrated a low publication bias probability with a significant *p* value of .541 for the Beggs test.^30^

### 3.3 Statistical analysis: Risk ratio (RR) of all included studies

**Figure 2:**
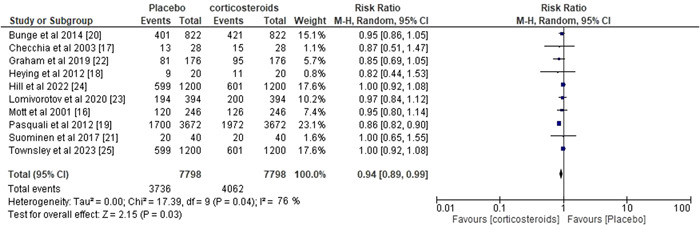
Forest plot risk ratio corticosteroids versus placebo.

All 10 trials, with a total of 7798 participants, provided information on effects of perioperative corticosteroids on children undergoing cardiac surgery. Figure 2 depicts the comparative assessment of corticosteroids versus placebo and the pooled RR was 0.94 with 95% CI of 0.89‐0.99, *I* ^2^ value of 76% and *p* = .03.

### 3.4 Statistical analysis of primary outcome: All‐cause mortality

**Figure 3:**
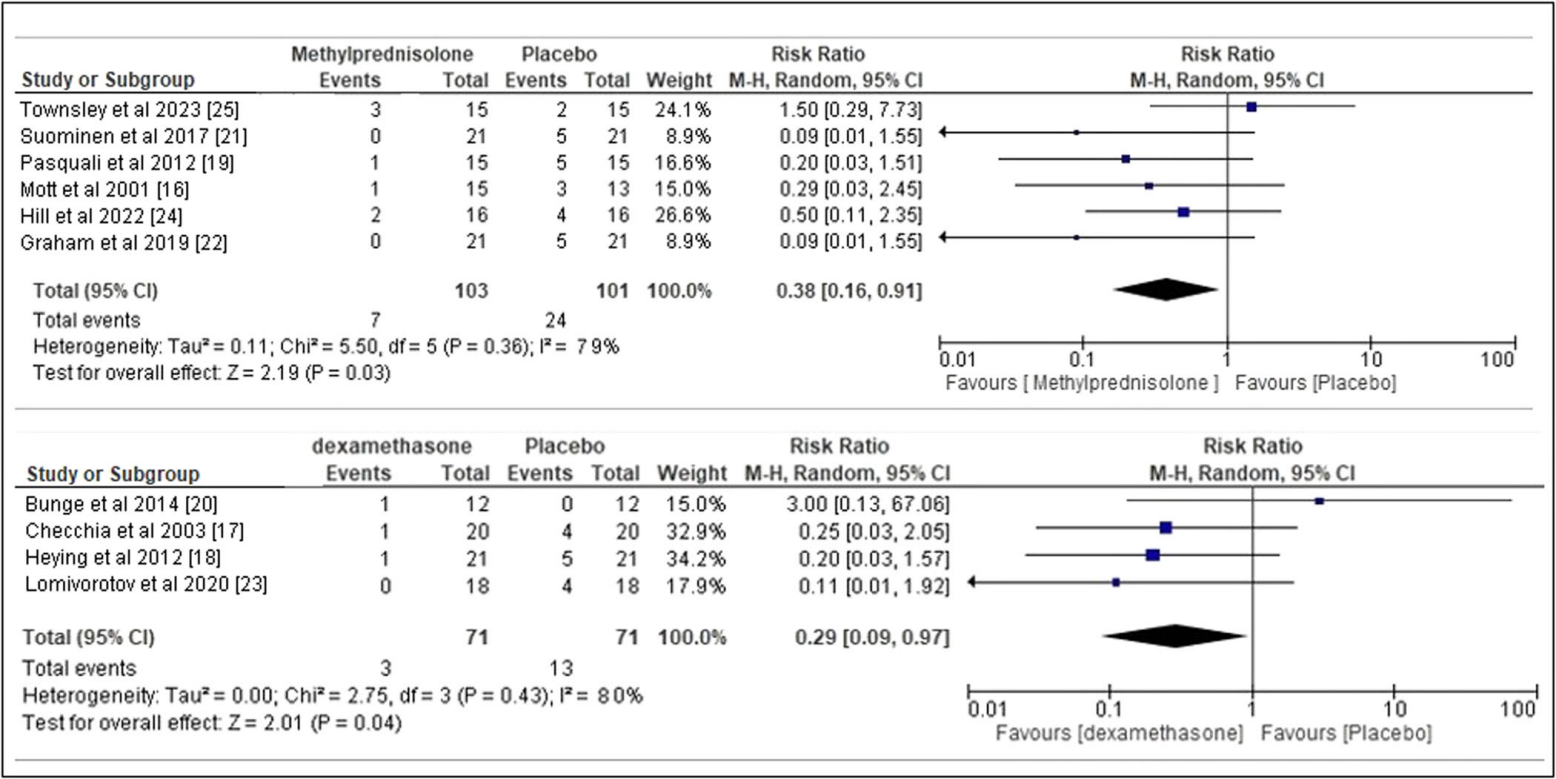
Forest plot risk ratio all‐cause mortality.

For all‐cause mortality, no significant difference was detected in the mortalities of the corticosteroid therapy group and the placebo group as illustrated in Figure 3. The pooled RR for Methylprednisolone was 0.38 with 95% CI of 0.16‐0.91, *I* ^2^ value of 79%, and *p =* .03 whereas the pooled RR for dexamethasone was 0.29 with 95% CI of 0.09−0.97, *I* ^2^ value of 80%, and *p* = .04.

### 3.5 Statistical analysis of secondary outcome: Length of hospital stay

**Figure 4:**
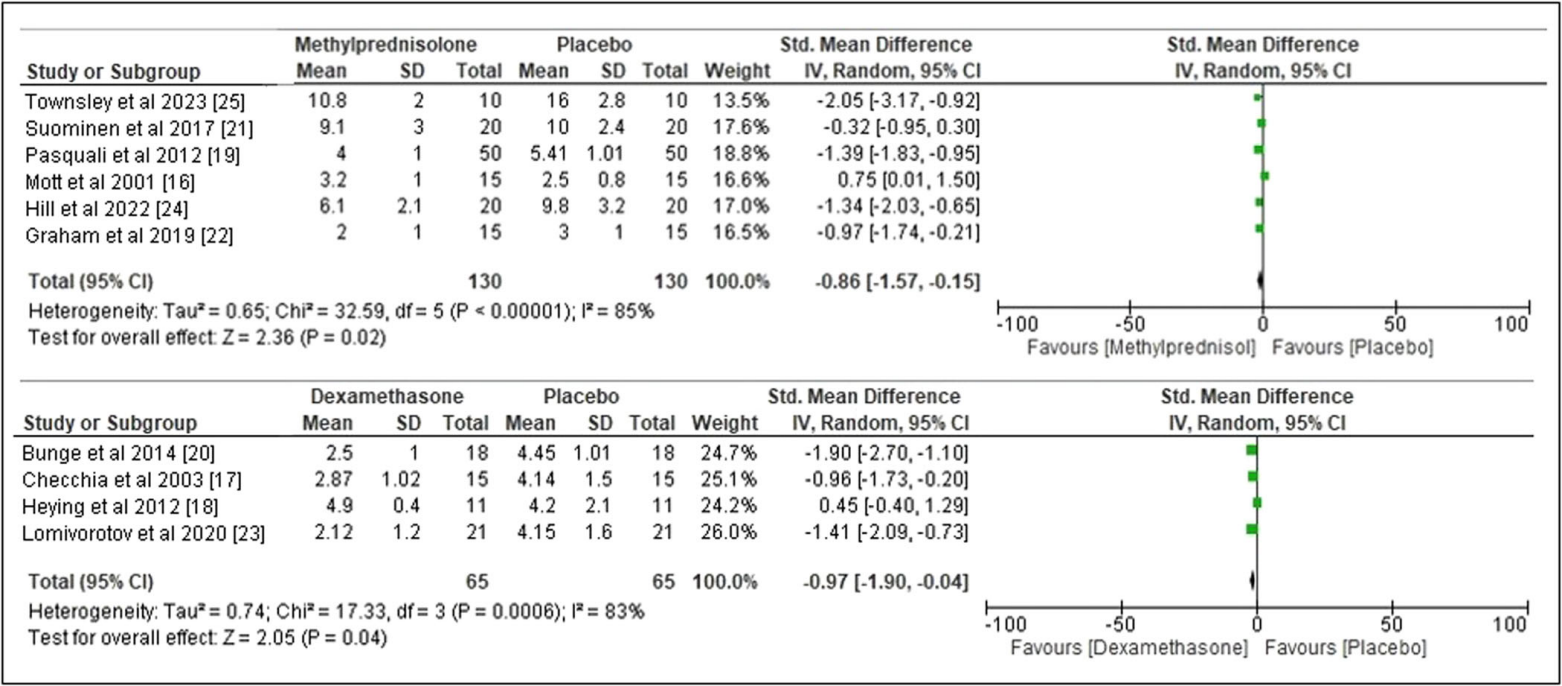
Forest plot risk ratio length of hospital stay.

For length of hospital stay, significant difference was detected in between the corticosteroid therapy group and the placebo group as illustrated in Figure 4. The pooled standard mean difference (SMD) for Methylprednisolone was −0.86 with 95% CI of −1.57 to −0.15, *I* ^2^ value of 85%, and *p* = .02 whereas the pooled SMD for corticosteroid dexamethasone was −0.97 with 95% CI of −1.90 to −0.04, *I* ^2^ value of 83%, and *p* = .04.

## 4 DISCUSSION

Corticosteroids have been used prophylactically in pediatric heart surgery for over 60 years, but their use is still debated. Corticosteroids are used in CPB Pediatric cardiac surgery for three reasons: to reduce the extracorporeal circuit‐induced systemic inflammatory response syndrome (SIRS), to offer perioperative supplementation for anticipated relative adrenal insufficiency; and for the presumed neuroprotective impact during profound hypothermic circulatory arrest surgeries.^31^, ^32^

Effects of perioperative corticosteroids were compared to placebo in 10 randomized controlled studies involving children undergoing cardiac surgery. Dexamethasone and methylprednisolone are the studied corticosteroids which are routinely administered in the included trials, beginning 4 hours before to the start of CPB and continuing for many days after surgery. Several substantial findings have evolved despite the fact that research have found varying results. Perioperative corticosteroids may not reduce primary outcomes which is the postoperative in‐hospital mortality from all causes. It occurred due to the high prevalence of SIRS such as ischemia‐reperfusion injury, endothelial cell infiltration, and so on in CPB patients.^33^, ^34^ Although corticosteroids given before surgery were linked to a shorter hospital stay, which was a secondary outcome.

Children were thought to be more at risk for this kind of SIRS because they experience more severe haemodilution, have a reduced circulation volume, and experience more challenging surgical procedures than adults.^35^, ^36^, ^37^, ^38^ Factually, corticosteroids have been both the most effective and most controversial therapeutic medications for preventing the SIRS associated with CPB. The above‐mentioned pathophysiologic processes as a result of CPB were, however, substantially relieved by the development of perfusion and postoperative treatment regimens, leading to a decrease in mortality and morbidity.^39^, ^40^ Hence, it was not known if or to what degree corticosteroids' direct inhibition of SIRS would lead to better clinical results.

The use of perioperative corticosteroids has not been linked to a lower risk of death in a retrospective study of 848 children undergoing heart surgery, but they may dramatically shorten the duration of the patient's stay in the hospital.^39^ A similar finding was reported in another retrospective study of neonatal patients.^41^, ^42^ While intraoperative corticosteroids were found to be very beneficial at one institution.^43^ Recent randomized controlled research of 232 patients found no statistically significant difference between corticosteroids and placebo in mortality, duration of mechanical ventilation and length of hospital stay.^44^ After congenital heart surgery, corticosteroids have been shown to improve cardiac function and reduce serum myocardial enzyme levels in a number of trials.

Our data suggested that corticosteroid treatment could shorten patients' stays in the hospital, but had no appreciable impact on mortality rates overall. The discrepancy in findings between studies highlights the need for more controlled experiments to determine if the observed pattern represents a true difference or merely an artefact of chance.

## 5 STUDY LIMITATIONS

Several limitations exist in this study. As a first step, we included trials using a wide range of corticosteroid doses, administration frequencies, and types; this suggested that a subgroup analysis could be necessary if there were enough participants. Second, a small study effect may have been at play because the bulk of the included studies had relatively small sample sizes. Third, the methylprednisolone dose used in this study (30 mg/kg) was higher than what is often used in clinical practice, which may have impacted the outcomes. Children of all ages who required cardiac surgery were included in this research. But, as is well‐known, neonates and older kids have quite distinct physiologies, therefore, the findings cannot be applied to the population as a whole.

## 6 CONCLUSION

In conclusion, preventive corticosteroids in cardiac surgery did not reduce mortality in the present meta‐analysis of 10 RCTs including 7798 participants, however, it reduces the duration of hospital stay. An upsurge in myocardial adverse events has not been fully elucidated in terms of its clinical implications. We showed that the administration of perioperative corticosteroids may not significantly improve the clinical outcomes of all‐cause mortality and length of hospital stay in children undergoing heart surgery. To draw definitive conclusions about the risk‐benefit profile of these perioperative corticosteroids, more data are urgently required from ongoing randomized controlled trials involving the administration of corticosteroids such as Dexamethasone and Methylprednisolone to reduce Systemic Inflammation after Neonatal and Pediatric Cardiac Surgery.

## AUTHOR CONTRIBUTIONS

*Concept and designed the study, analyzed data and drafting of the manuscript*: Daliu Chen. *Collected the data and helped in data analysis, proofreading and final editing along with guarantor of the manuscript*: Yongchun Du. All authors read and approved the final version of the manuscript.

## CONFLICT OF INTEREST STATEMENT

The authors declare no conflict of interest.

## Supporting information

Supplementary Figure 1.

Supplementary Figure 2.

Supplementary Figure 3.

## Data Availability

All data generated or analyzed during this study are included in this article. Further enquiries can be directed to the corresponding author.
