# Supplementary figures and images for: Analysis of perioperative corticosteroid therapy in children undergoing cardiac surgery: A systematic review and meta‐analysis
Source: Clin Cardiol. 2023 Apr 26;46(6):607–14. doi: 10.1002/clc.24018 (PMC10270253; doi:10.1002/clc.24018)

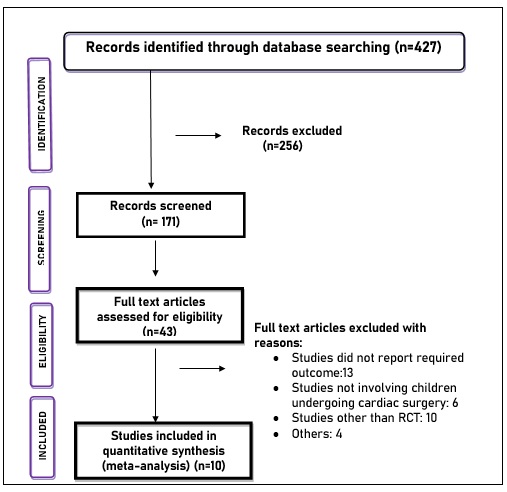

Supplement: Supplementary file 1 — Supplementary Figure 1. [file CLC-46-607-s003.jpg]

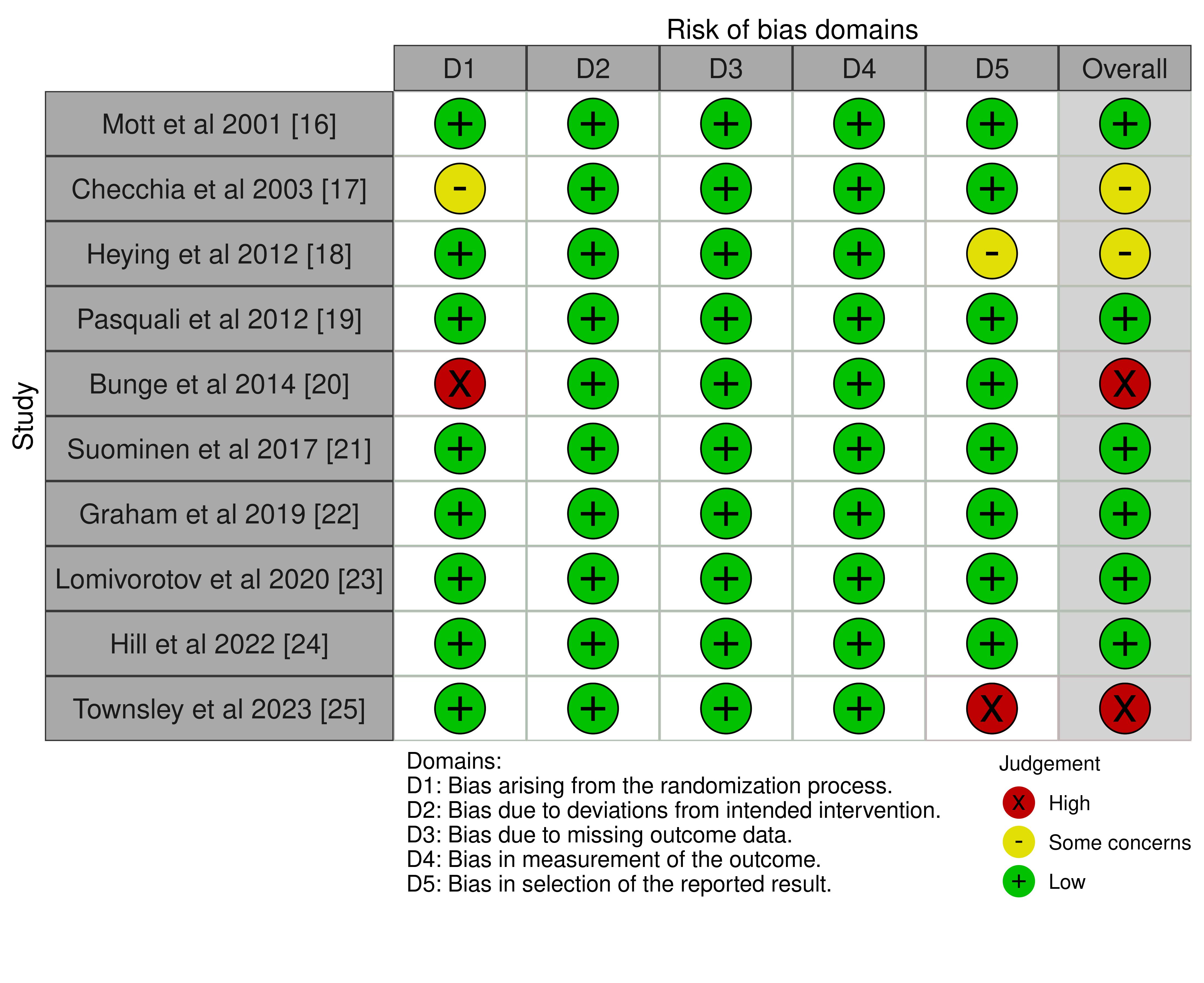

Supplement: Supplementary file 2 — Supplementary Figure 2. [file CLC-46-607-s002.jpeg]

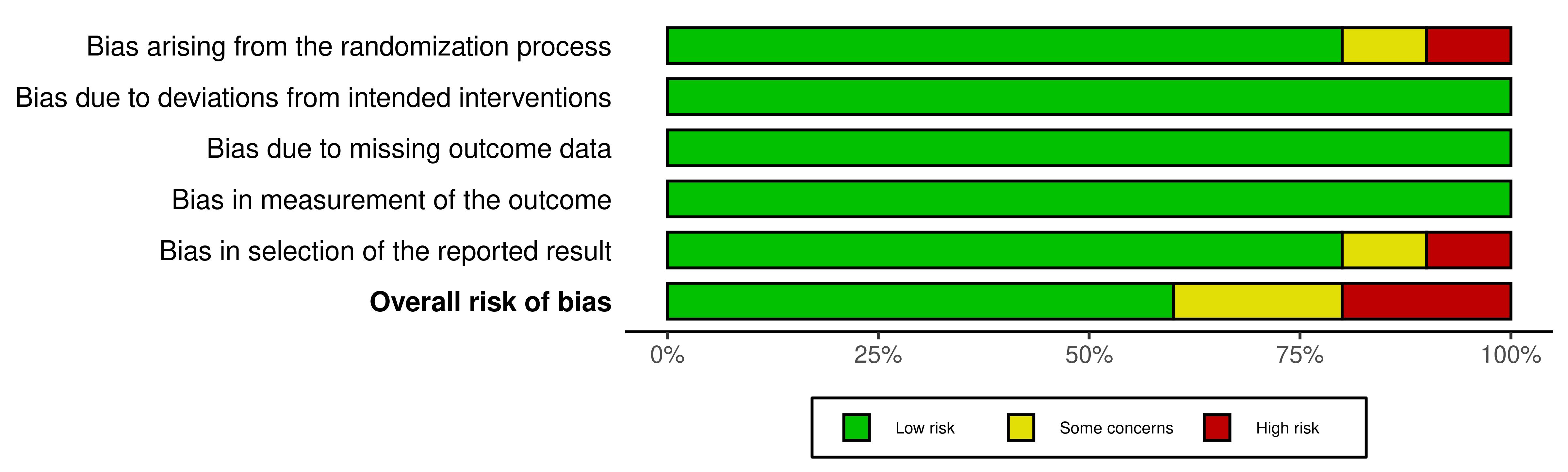

Supplement: Supplementary file 3 — Supplementary Figure 3. [file CLC-46-607-s001.jpeg]
